# Editorial: Interference of COVID-19 and Influenza Infections

**DOI:** 10.3389/fpubh.2021.818199

**Published:** 2021-12-23

**Authors:** Lin Yang, Daihai He, Lin Wang

**Affiliations:** ^1^School of Nursing, Hong Kong Polytechnic University, Kowloon, Hong Kong SAR, China; ^2^Department of Applied Mathematics, Hong Kong Polytechnic University, Kowloon, Hong Kong SAR, China; ^3^Department of Genetics, Faculty of Biology, University of Cambridge, Cambridge, United Kingdom

**Keywords:** COVID-19, influenza, interference, vaccine, co-infected patients, severity

All the previous pandemics in the past century were caused by influenza viruses (1), whereas the ongoing COVID-19 pandemic is the first pandemic in documented history that was caused by a coronavirus. Coronavirus and influenza virus share many common features. Both are respiratory RNA virus, attack several host species, evolve rapidly (2), and have comparable epidemiological characteristics (3). The goal of this Research Topic is to encourage the laboratory, epidemiological, and modeling studies on the potential interference of COVID-19 and influenza viruses, at both the individual and population levels.

Dadashi et al. did a systematic review and meta-analysis on co-infection of COVID-19 and influenza (4). The proportion of COVID-19 patients with influenza co-infection was generally low, with a pooled estimate 0.8% (95% CI: 0.4–1.3%). Such proportions had great variations across countries, gender, and age groups (<50 yrs vs. ≥50 yrs). However, the testing capacities for influenza could have been rather limited in some places amid the COVID-19 pandemic. This could lead to underestimated co-infection rates. Another research gap identified from this review was that, none of the studies had compared the clinical severity and outcomes between COVID-19 patients with and without influenza co-infection, and only the case reports/case series studies were included in the review. Geng et al. reported the incidence of influenza and tuberculosis cases in a tertiary hospital in Northern China, and found the test positive proportion of influenza was significantly lower in 2020 than in the pre-pandemic period (5). Their finding echoes the surveillance data from other countries, which also reported low influenza virus activities in the COVID-19 pandemic. As pointed out by the authors, non-pharmaceutical interventions and changed healthcare seeking behavior might be the reasons behind the suppressed seasonal influenza epidemics. Interestingly, no obvious reduction was found in the test positive proportion of *Mycobacterium tuberculosis*, a bacterium that primarily adopts airborne transmission. A more comprehensive overview about the change of other respiratory viruses and underlying biological mechanisms is still needed.

At the beginning of the COVID-19 pandemic, numerous research efforts previously made on seasonal and pandemic influenza have provided solid evidence on the prevention and control strategies against the COVID-19. By adapting a questionnaire that was originally designed on the attitude about influenza vaccine, Cai et al. quickly conducted an online survey in 1,057 Chinese adolescents about their attitude and acceptance toward COVID-19 vaccines, when these vaccines became available but not licensed for use in adolescents yet (6). They found that 75.59% of respondents were willing to accept COVID-19 vaccines, which was similar to the acceptance rates among adults and parents reported elsewhere. Vaccine acceptance in teenagers is associated with knowledge on COVID-19 vaccine, perceived efficacy, and peer support from family members and friends. Unfortunately, the authors did not ask the participants about their influenza vaccination history and perceived severity on COVID-19, and no data from their parents/guardians who usually make the decision of vaccination. These potential vaccination barriers shall be worthy of further investigations in the future studies. Tao et al. reported another online survey in 3,011 Chinese women of childbearing age (aged 18–49 years) (7). The acceptance rate for both COVID-19 and influenza vaccines was 82.1% among these participants, although only 27.7% of them reported a history of influenza vaccination. It is interesting to note that women with pregnancy (or parity) experience had a lower acceptance rate for both COVID-19 and influenza vaccines than those without such experiences. This could explain the trend of decreasing acceptance rates with age. The study design did not allow the authors to check the pregnant status of each participant, whereas pregnant women are expected to have high vaccination hesitancy due to limited clinical trials in this vulnerable group.

Recently, two oral antivirals have shown effectiveness in reduction of hospitalization and deaths among moderate to severe COVID-19 patients. With more and more people around the world get vaccinated, it is time to discuss the optimal strategies of relaxing social distancing measures and resuming normal life. Xiao et al. compared the different strategies of reopening pathways in the scenario of Ontario, Canada (8). Their models suggest that the relaxation of control measures need to be based on careful evaluation of vaccination coverage, healthcare capacities, as well as emerging variants of concern. The work done by Xiao et al. could be extended to other regions/countries and potential developed to provide the real-time simulations and feedbacks. It is not an easy task but surely these could be what the policy makers are truly keen to get.

Bouba et al. conducted an ecological study to investigate the association of economic development and health service indicators with COVID-19 incidence and deaths at the national level in 54 African countries (9). They found that countries with more testing capacities, poorer health service capacities and higher Gini indexes had higher incidence rates of COVID-19 cases. But surprisingly, they also found the number of nurses was associated with higher risks of deaths from COVID-19, which needs further investigations. The deaths of COVID-19 could have been seriously underestimated in the African countries with limited testing capacities and health services. The ecological fallacy and measurement bias might be the reasons to derive this controversial association, as recognized by the authors. Although the authors did not assess the association of influenza cases with the disease burden of COVID-19, we could speculate that health and economic inequality could similarly enhance the adverse effects of seasonal influenza on population health. At present, a large proportion of people in most African countries remain unvaccinated for COVID-19, who should not be ignored. The SARS-CoV-2 may keep circulating in these low-resource countries and spread to the other countries where more people get vaccinated. Even in the post-pandemic era, the WHO and developed countries shall allocate more resources to these countries, not only for the COVID-19 vaccines but also for influenza vaccines, as both viruses could be circulating in the human populations for quite a long time.

We appreciate the important research work done by the authors who contributed to this Research Topic. It has been noted that influenza virus remained at low levels in the past 2 years, largely due to stringent social distancing measures adopted in many countries. It remains a concern whether seasonal epidemics of influenza will appear again and merge with the COVID-19 peaks in the post-pandemic era.

## Author Contributions

LY drafted the manuscript. All authors approved the final version.

## Funding

LY is supported by the HMRF Commissioned Research on the Novel Coronavirus Disease (COVID-19) (COVID1903007). DH is supported by the Alibaba (China) Collaborative Fund.

## Conflict of Interest

DH was supported by the Alibaba China Collaborative Fund. The remaining authors declare that the research was conducted in the absence of any commercial or financial relationships that could be construed as a potential conflict of interest.

## Publisher's Note

All claims expressed in this article are solely those of the authors and do not necessarily represent those of their affiliated organizations, or those of the publisher, the editors and the reviewers. Any product that may be evaluated in this article, or claim that may be made by its manufacturer, is not guaranteed or endorsed by the publisher.
